# Age-associated methylation change of *CHI* promoter in herbaceous peony (*Paeonia lactiflora* Pall)

**DOI:** 10.1042/BSR20180482

**Published:** 2018-09-14

**Authors:** Yanqing Wu, Lei Liu, Daqiu Zhao, Jun Tao

**Affiliations:** 1 College of Animal Science and Technology, Yangzhou University, Yangzhou 225009, P.R. China; 2 Key Laboratory of Crop Genetics and Physiology of Jiangsu Province, College of Horticulture and Plant Protection, Yangzhou University, Yangzhou 225009, P.R. China

**Keywords:** CHI gene, expression, methylation, Paeonia lactiflora

## Abstract

Chalcone isomerase gene (*CHI*) is a key gene that regulates the formation of yellow traits in petals. To reveal transcriptional regulatory mechanisms of *CHI* gene in petals of *Paeonia lactiflora*, we investigated the *CHI* expression using qPCR, the pigment content by HPLC, and methylation levels using BSP+Miseq sequencing in ‘Huangjinlun’ variety during different developmental stages including flower-bud stage (S1), initiating bloom (S2), bloom stage (S3), and withering stage (S4). Results showed that the expression level of *CHI* gene at S2 stage was significantly higher than that at other stages (*P*<0.05), and at S4 stage was extremely significantly lower than other stages (*P*<0.01). Besides, total anthocyanin, anthoxanthin, and flavonoid contents in petals presented a similar trend with *CHI* expression during developmental stages. A total of 16 CpG sites varying methylation levels were detected in *CHI* gene core promoter region, of which the methylation levels at mC-4 and mC-16 sites were extremely significantly negatively correlated with *CHI* mRNA expression (*P*<0.01). mC-16 site is located in the binding region of C/EBPα transcription factor, suggesting that methylation at the mC-16 site may inhibit the binding of C/EBPα to *CHI* promoter DNA, thereby regulating the tissue-specific expression of *CHI* gene. Our study revealed the expression pattern of *CHI* gene in petal tissues of *P. lactiflora* at different developmental stages, which is related to promoter methylation. Moreover, the important transcription regulation element–C/EBPα was identified, providing theoretical reference for in-depth study on the function of *CHI* gene in *P. lactiflora*.

## Introduction

Herbaceous Peony (*Paeonia lactiflora* Pall.) is a traditional Chinese flower, and its color quality directly influences the ornamental merit and commercial value. There are the rich resources of *P. lactiflora* varieties with different colors including pink, red, and purple in China; however, only one variety ‘Huangjinlun’ has yellow flower. Therefore, the cultivation of new *P. lactiflora* varieties with novelty colors such as yellow is currently an important project for ornamental plant breeders. At present, flower pigmentation is caused by the accumulation of pigments within the epidermal cells, of which yellow pigments are mainly composed of flavonoids and carotenoids. In the flavonoid biosynthetic pathway, the formation of yellow pigments is related to chalcone isomerase (*CHI*) gene [1]. *CHI* is a very stable enzyme participating in the early stage of flavonoid biosynthesis, and greatly accelerating the intramolecular cyclization of chalcones to form the flavonones. The activity of *CHI* enzyme is necessary for the biosynthesis of flavanone precursors and phenylalanine phytoalexins in the synthesis of anthocyanins [2]. Therefore, the *CHI* gene plays a very important role in the development of yellow flowers. Previous studies revealed that the expression levels of *CHI* gene directly affected the accumulation of upstream yellow chalcone, the downstream colorless or yellowish anthocyanins and red anthocyanins, leading to the changes in colors or flavonoids. In petunia mutants, the *CHI* expression was decreased because of the mutation of *CHI* promoter, resulting in the formation of yellow or green pollen [3]. The decreased expression of *CHI* gene in *Cyclamen persicum* led to the accumulation of abundant chalcone to produce yellow flowers [4]. A loss-of-function mutation of *CHI* gene based on transposon insertion resulted in forming the yellow flowers of *Dianthus caryophyllus* [5]. In *Allium cepa*, inactivation of *CHI* gene led to the reduction in flavonoid quercetin content [6]. Therefore, in order to investigate whether the expression level of *CHI* gene is related to the formation of petal yellow, we used *P. lactiflora* variety ‘Huangjinlun’ to examine the differential expression of *CHI* gene from different developmental stages at flower-bud stage (S1), initiating bloom (S2), bloom stage (S3), and withering stage (S4) for better understanding the *CHI* gene expression patterns in *P. lactiflora* petals.

DNA methylation in the promoter region is one of the major epigenetic modifications in eukaryotic genomes. In eukaryotes, methylation occurs only in the fifth carbon atom of cytosine, and the reaction is catalyzed by DNA methyltransferase to transfer S-adenosylmethionine (SAM) as methyl donor to cytosine, leading to the formation of 5-methyl cytosine [7]. DNA methylation may exist in all higher organisms where 60–90% of the GC sequences in the genome are methylated, but the proportion of methylated DNA in the whole genome is usually small. Methylated cytosine contents are greatly different among organisms, such as nematodes without methylated cytosine, mammals and birds with ~5% methylated cytosine, fish and amphibians with ~10% methylated cytosine, plant species with more than 30% methylated cytosine etc. [8]. DNA methylation existed in certain differences among different tissues or different development stages in a particular organism [9]. Therefore, DNA methylation distribution is species-specific and tissue-specific, varying with different development stages [10].

At present, the traditional methods for quantitative detection of methylation level include Sanger sequencing and pyrosequencing. The Sanger sequencing method has some limitations including poor quantitative accuracy caused by the limited number of selected clones and sample differences among clones selected from different batches, and the larger time-consuming and labor-intensive workload [11]. Pyrosequencing offers a protocol of quantifying methylation level by detecting fluorescence values, but is also restricted to the disadvantage of low accuracy, especially when hypermethylation or hypomethylation is occurred and read sequence length (usually no more than 100 bp) is relatively shorter for completely covering the CpG island region [12]. The Illumina MiSeq v4 PE300 benchtop sequencer has now reached 2 × 300 bp in length, allowing most of the CpG islands to be covered [13]. In brief, Miseq sequencing is superior to the Sanger sequencing and pyrosequencing methods for the quantitative detection of methylation in the region of interest. In previous study, we obtained the upstream promoter sequence of *CHI* gene using RACE cloning and chromosome walking and determined the core region (–1651 to –2050 bp) of *CHI* promoter through the combination analysis of CpG island prediction and dual-luciferase assay [14]. In the present study, we used BSP + Miseq sequencing method [15] to quantitatively detect the methylation level of CpG island in the core promoter region of *CHI* gene in *P. lactiflora* and analyzed the effects of important methylated sites and transcription factors on *CHI* mRNA expression. The results further revealed the expression regulatory mechanism of *CHI* gene, providing theoretical guidance for further in-depth verification of *CHI* gene function in *P. lactiflora*.

## Materials and methods

### Experimental plants

**Figure 1:**
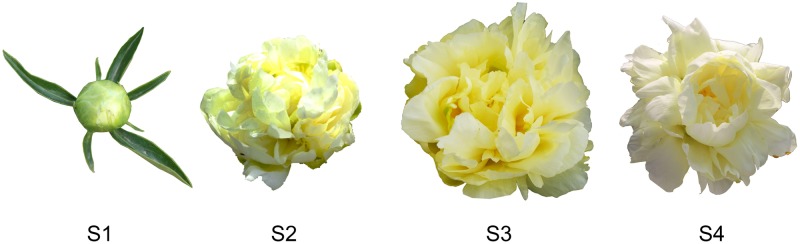
Petal changes at different stages of *P. lactiflora* S1, S2, S3, S4 represent the flower-bud, initiating bloom, bloom and withering stages, respectively.

In the present study, 32 petal tissues of *P. lactiflora* variety ‘Huangjinlun’ were collected from the germplasm repository of Horticulture and Plant Protection College, Yangzhou University, Jiangsu Province, P.R. China (32°30′N, 119°25′E) at four different development stages: flower-bud stage (S1, *n*=8), initiating bloom (S2, *n*=8), bloom stage (S3, *n*=8), and withering stage (S4, *n*=8) (Figure 1). Petal samples were quickly frozen by liquid nitrogen, then stored in freezer at –80°C.

### Primer design

cDNA sequence of *P. lactiflora CHI* gene (Accession: JN119872.1) from our previous study [14] was used to design specific expression primers using Primer5.0 software (Table 1). *P. lactiflora β-actin* gene (JN105299) was used as the internal reference. All the primers were synthesized by Shanghai Biological Engineering Services Limited.

**Table 1:** *CHI* primer information

Primer	(5′-3′ sequence)	Target
*CHI*	F: TCCCACCTGGTTCTTCTA	qRT-PCR
	R: AACTCTGCTTTGCTTCCG	
*β-Actin*	F: GCAGTGTTCCCCAGTATT	qRT-PCR
	R: TCTTTTCCATGTCATCCC	

### Total RNA extraction and cDNA synthesis

Total RNA of *P. lactiflora* was extracted by Trizol method. The isolated RNA was dissolved in DEPC water, and then stored at –80°C. cDNA synthesis system (10 μl) includes 1 μl of total RNA, 1 μl of 3′ RACE adapter (5 μM), 2 μl of 5× M-MLV buffer, 1 μl of dNTP mixture (10 mM each), 0.25 μl of RNase inhibitor, 0.25 μl of reverse transcriptase M-MLV (200 U/μl), and 4.5 μl of RNase-free ddH_2_O. The reaction conditions were carried out with 42°C for 60 min and 70°C for 15 min. After the reaction was completed, the reaction solution was stored at –20°C.

### qPCR analysis

The reverse transcribed cDNA was used as template for fluorescence quantitative PCR reaction. The reaction system (25 μl) was composed of 12.5 μl of SYBR® Premix Ex Taq™ (2×), 0.5 μl of PCR forward primer, 0.5 μl of PCR reverse primer, and 0.5 μl of Rox Reference Dye II (50×), 2 μl of cDNA template, and 9 μl of ddH_2_O. The reaction conditions were carried out as follows: pre-denaturation at 95°C for 5 min, followed by 40 cycles of denaturation at 95°C for 15 s, annealing at 60°C for 15 s, and extension at 72°C for 40 s. PCR products were detected by 1% agarose gel electrophoresis.

### Qualitative and quantitative analysis of flavonoids

Petals of each sample (1.0 g fresh weight) were extracted with 6 ml of acidic methanol solution (70:0.1:29.9; v/v/v, CH_3_OH: HCl: H_2_O) at 4°C for 24 h. Qualitative and quantitative analysis of flavonoids was performed using HPLC-ESI-MS^n^ (LCQ Deca XP MAX, Thermo) coupled with photodiode array and mass spectrometry detectors (HPLC-PDA-MS, Thermo company) with a three-dimensional quadrupole ion trap mass spectrometer. The HPLC column was TSK gel ODS-80Ts QA (4.6 mm × 250 mm) (Tosoh, Japan). The specific conditions were the same as the report of Zhao et al. [16] with some modifications. Each peak area of anthocyanins and anthoxanthins was detected under 525 and 350 nm was recored. Additionally, total flavonoid contents were determined as the sum of all anthocyanins and anthoxanthins.

### PCR amplification of *CHI* core promoter and prediction of important transcription factor binding sites

Based on the upstream promoter sequence of *CHI* gene from *P. lactiflora* we previously obtained [14], nested PCR was used to amplify core promoter region of *CHI* gene with the forward primer F: GACTCTGTGCTGAGAGTAGTAGTAAG, and the reverse primer R: CTGTGAGTCAGTAGGAAATTGATGTG at the annealing temperature of 57°C. The transcription factor binding sites of *CHI* core promoter was predicted using Alibaba 2 software (http://gene-regulation.com/pub/programs/alibaba2/index.html).

### Methylation sequencing

Genomic DNA was extracted from tissues by standard phenol/chloroform extraction and subjected to bisulfite conversion using the EpiTect bisulfite kit (Qiagen, Valencia, CA, U.S.A.) according to the manufacturer’s instructions. Touchdown PCR was used to amplify the bisulfite-treated DNA (BST-DNA) using the following *CHI* primer sequences: forward, 5′-GGGATTTGGTAGATTTTTATAGTTTA-3′ and reverse, 5′- AACTCTCCCAACAATACAAACACTC-3′. The NGS library was built according to the TruSeq DNA PCR-free library construction instruction (Illumina, San Diego, CA, U.S.A.) and sequenced with the Illumina MiSeq benchtop sequencer. Illumina Experiment Manager (Illumina, San Diego, CA, U.S.A.) was used to generate FASTQ format files. A two-tailed end sequencing library was performed with 600 cycle MiSeq v.3 reagent cartridges (Illumina, San Diego, CA, U.S.A.). The original sequencing data are cleaned to remove low quality and blurry base sequences for quality assurance, and then used to calculate methylated and non-methylated reads. Finally, methylated and unmethylated reads were combined to calculate the methylation of each of CpG sites as the levels of the methylated reads.

### Data statistical analysis

Gene relative expression levels of *CHI* gene were calculated by the 2^−ΔΔ*C*^_t_ comparative threshold cycle (*C*_t_) method [17]. Single-factor ANOVA was conducted using SPSS16.0 software to analyze the differences in expression levels of *CHI* gene among different developmental stages. Meanwhile, Pearson’s method was used to estimate the correlation between methylation levels at different CpG sites and gene expression levels.

## Results and analysis

### Developmental expression pattern analysis of *CHI* gene in *P. lactiflora* variety ‘Huangjinlun’

**Figure 2:**
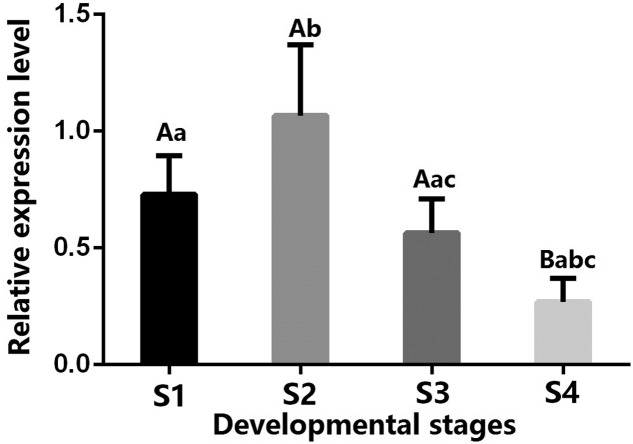
Expression patterns of *CHI* gene in the petals from different developmental stages in *P. lactiflora* variety ‘Huangjinlun’ S1, S2, S3, and S4 represent the flower-bud, initiating bloom, bloom, and withering stages, respectively.

In the present study, qPCR was used to detect the differential expression of *CHI* gene at different developmental stages in *P. lactiflora* cultivar ‘Huangjinlun’. As shown in Figure 2, the expression level of *CHI* gene in petals of initiating bloom (S2) was significantly higher than that at flower-bud stage (S1) and bloom stage (S3) (*P*<0.05), and was extremely significantly higher than that at withering stage (S4) (*P*<0.01). The gene expression of *CHI* gene in petals of S4 was the lowest, and extremely significantly lower than that at S1, S2, and S3 stages, respectively (*P*<0.01).

### Identifcation of flavonoid composition

Flavonoids, including anthocyanins and anthoxanthins, each have a different ultraviolet absorption wavelength. The results revealed the presence of similar compounds among four different groups including flower-bud stage (S1), initiating bloom (S2), bloom stage (S3), and withering stage (S4), although their peak areas varied in both anthocyanin and anthoxanthin chromatograms (Supplementary Figure S1). Based on retention time, ultraviolet–visible spectral properties, mass spectrometric data and main fragmentations, eight main flavonoids, including two anthocyanins and six anthoxanthins, were separated and characterized (Supplementary Table S1). Total anthocyanin, anthoxanthin, and flavonoid contents in petals basically presented a downward trend with flower development, reaching a maximum in S2 and a minimum in S4 (Table 3). Besides, the content of flavonoids was significantly correlated with *CHI* mRNA expression during developmental stages (*P*<0.05).

### Methylation detection of *CHI* core promoter in *P. lactiflora*

**Figure 3:**
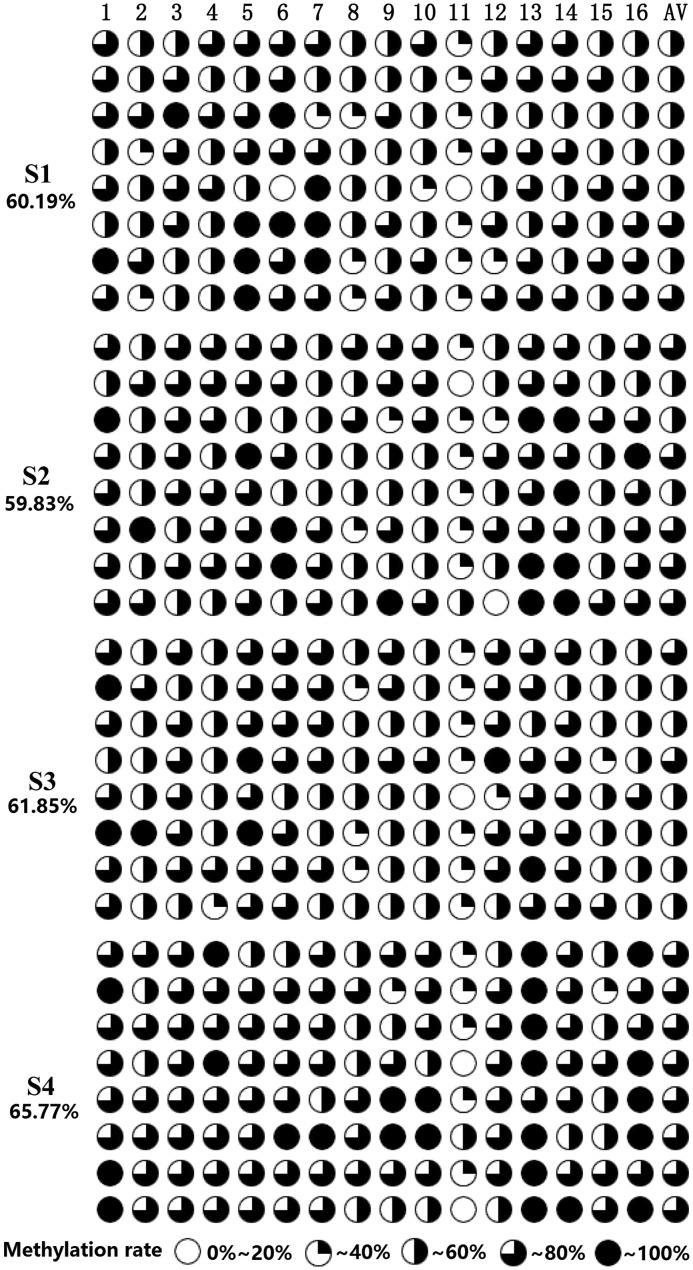
Frequency of DNA methylation of 5-mC sites in *P. lactiflora* petals from different stages S1, S2, S3, and S4 represent the flower-bud, initiating bloom, bloom, and withering stages, respectively. CpG sites are marked with pie charts, black regions represent methylation level; CpG sites are marked with pie charts

**Figure 4:**
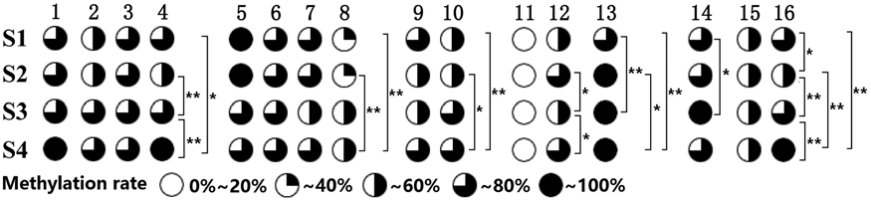
*CHI* gene methylation levels in petals of *P. lactiflora* at different developmental stages CpG sites are marked with pie charts, black regions represent methylation level; CpG sites are marked with pie charts; * within the same CpG site means different significantly (*P*<0.05); ** within the same CpG site means extremely different significantly (*P*<0.01).

**Figure 5:**
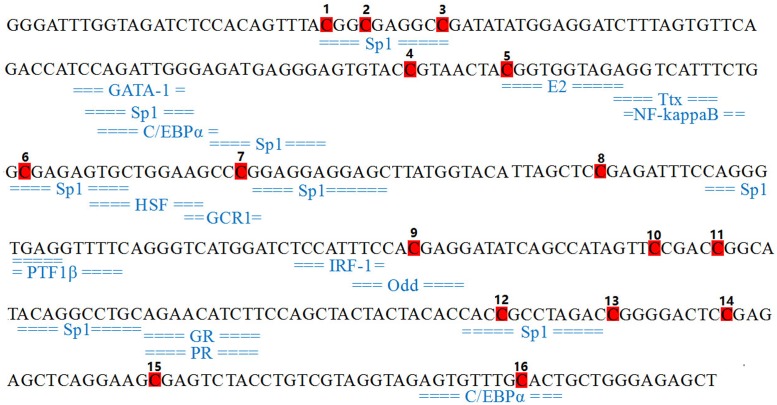
Identification of putative transcription factor binding sites (TFBS) in CpG islands of *CHI* gene promoter in *P. lactiflora* Shaded sequence, CpG site; underlined sequence, TFBS.

Nested PCR amplification products were sequenced to identify core promoter sequence of *CHI* gene in *P. lactiflora* variety ‘Huangjinlun’ (Figure 5). A total of 16 CpG sites were methylated in amplification fragments of *CHI* gene. Overall, methylation levels ranged from 59.8% to 65.7% at different developmental stages; however, the degree of dispersion in single methylation site was relative higher (Figure 3 and Supplementary Table S2). Methylation levels at mC-4, mC-8, mC-10, mC-12, mC-13, mC-14, and mC-16 sites showed significant (*P*<0.05) or extremely significant (*P*<0.01) differences among different developmental stages. Above all, there existed the greatest difference at mC-16 site (Figure 4).

### Correlations between methylation levels and mRNA expression of *CHI* gene in *P. lactiflora* petals from different developmental stages

Pearson correlation analysis was performed between methylation levels and mRNA expression of *CHI* gene in petal tissues from different developmental stages (S1, S2, S3, and S4). As shown in Table 2, the methylation levels of most CpG sites in the amplified fragments of *CHI* gene were negatively associated with the mRNA expression levels, among which, the corrections between methylation levels at mC-4, mC-16 sites and mRNA expression were extremely significantly negative (*P*<0.01).

**Table 2:** Correlation analysis between methylation level and mRNA expression in *CHI* gene

CpG site	Correlation coefficient	*P*-value
CpG_1	–0.031	0.868
CpG_2	0.011	0.952
CpG_3	–0.117	0.524
CpG_4	–0.474	0.006
CpG_5	0.058	0.752
CpG_6	–0.210	0.248
CpG_7	–0.208	0.254
CpG_8	–0.319	0.075
CpG_9	–0.116	0.528
CpG_10	–0.232	0.201
CpG_11	0.132	0.470
CpG_12	–0.281	0.120
CpG_13	–0.371	0.077
CpG_14	–0.012	0.950
CpG_15	–0.039	0.833
CpG_16	–0.682	0.001

**Table 3:** Flavonoid contents in *P. lactiflora* petals during flower development (μg g^−1^ FW)

Peak	S1	S2	S3	S4
a1	8.41 ± 0.35	9.85 ± 0.98	7.54 ± 0.0.39	5.83 ± 0.47
a2	137.42 ± 4.13	157.49 ± 6.77	122.01 ± 7.41	101.79 ± 5.65
f1	83.97 ± 3.18	96.95 ± 2.93	75.71 ± 4.19	64.69 ± 2.49
f2	2447.19 ± 69.18	2778.94 ± 82.19	2224.98 ± 64.31	1991.12 ± 99.19
f3	139.77 ± 8.87	167.90 ± 9.67	123.86 ± 4.33	104.25 ± 6.64
f4	116.73 ± 9.53	133.91 ± 6.76	111.93 ± 5.25	99.37 ± 5.62
f5	770.78 ± 30.41	880.56 ± 35.97	673.79 ± 37.89	591.83 ± 39.36
f6	768.09 ± 44.43	853.32 ± 42.59	656.16 ± 35.39	574.55 ± 36.56
Total anthocyanins	145.83 ± 4.33	167.34 ± 6.66	129.55 ± 7.25	107.62 ± 5.83
Total anthoxanthins	4326.53 ± 116.07	4911.59 ± 137.56	3866.42 ± 49.61	3425.81 ± 146.50
Total flavonoids	4472.36 ± 115.96	5078.93 ± 139.65	3995.97 ± 49.13	3533.43 ± 142.31

S1, S2, S3, and S4 represent the flower-bud, initiating bloom, bloom, and withering stages, respectively. a1–a2 indicate identified anthocyanins; f1–f6 indicate identified anthoxanthins.

### Determination of important transcription factors

In the present study, the transcription factor binding sites were predicted by using Alibaba 2 software. Figure 5 showed the transcription factors located within CpG sites in the core promoter region of *CHI* gene, including Sp1, E2, GCR1, Odd, and C/EBPα. Interestingly, no binding site was detected in mC-4, whereas mC-16 was located in the C/EBPα transcription factor binding site.

## Discussion

*CHI*, as the second known flavonoid biosynthesis-related enzyme, is one of the key enzymes required for the biosynthesis of flavonoids. The expression of *CHI* is directly related to the synthesis of anthocyanidins. Therefore, the transgenic research of *CHI* has been becoming one of the hot topics in genetic engineering of ornamental plants. Studies have shown that the lack of *CHI* gene expression or activity could seriously affect the flavonoid biosynthesis pathway in many plants, leading to a significant decrease in anthocyanidin and flavonoid contents [6,18], whereas the overexpression of *CHI* gene could increase flavonoid content [19]. Besides, the expression pattern of *CHI* gene has the characteristic of developmental-specificity, tissue-specificity, and spatial-specificity. In *Vitis vinifera*, the *CHI* gene is expressed in young leaves, young roots, pericarp, pulp and seeds, but different in different tissues. The *CHI* gene was highly expressed in the pericarp 30 and 90 d, in the flesh 90 d, and in the seed 45 d after anthesis [20]. Wang et al. [21] reported that the expression of *CHI* in the peel was decreased gradually during fruit maturation of ‘Guoqing No. 4’ satsuma mandarin (Citrus unshiu Marcow), but increased to a certain degree in the pulp. During the bud development of Camellia nitidissima, the expression of *CHI* gene was sharply increased first and then decreased gradually, indicating that the *CHI* gene was mainly expressed in the early stage of bud development of Camellia nitidissima. The *CHI* gene was expressed in bracts, sepals, petals, stamens and pistils, but the highest expression was detected in the pistils, followed by the stamens [22]. In the present study, we investigated the expression of *CHI* gene in petals of different developmental stages in *P. lactiflora* variety ‘Huangjinlun’, showing differences in the expression levels of *CHI* among four different developmental stages. There existed a certain significant difference in expression levels at four different stages, which may be due to the fact that the petal color of *P. lactiflora* gradually becomes lighter with the extension of stage [16]. Flower color was mainly dependent on the kinds of pigment, the content, and its distribution in the petals [23]. Previous studies on the determination of *P. lactiflora* petal pigment composition showed that a great deal of chalcone and a small amount of anthoxanthin accumulation were the cause of its yellow petal formation [2,24]. In the present study, we further detected the anthoxanthins content in *P. lactiflora* petals during different development stages and showed the significant correlation between anthoxanthins content and *CHI* expression. In *P. lactiflora*, the low-expression level of *CHI* induced most of the substrate accumulation in the form of chalcones and displaying yellow, changing a small part of substrates to anthoxanthins. Therefore, the expression level of *CHI* had direct association with the petal color formation of *P. lactiflora* during different development stages.

In order to further explore the regulatory mechanism of *CHI* gene expression in *P. lactiflora*, we analyzed the methylation levels of the core promoter region of *CHI* gene in petal tissues from different development stages by pyrosequencing and showed different methylation levels in 16 CpG sites. Studies have shown that the methylation of some key CpG sites in per gene promoter regions would result in changes of gene expression [25]. In the present study, we identified that the methylation levels at mC-4 and mC-16 sites in petal tissues of different developmental stages were extremely significantly correlated with *CHI* mRNA expression, whereas the mC-16 site was located in the transcription factor C/EBPα-binding site. Studies have shown that CpG sites were located in specific binding sites of some transcription factors, when these sites were methylated, the binding efficiency of the transcription factor to the promoter was reduced, leading to the decreasing of the gene transcription rate. Methylation-dependent transcription factors include Sp1 [26–28], CREB [29], USF-1 [30], CTCF [31], GATA-1 [32], AP-2 [33], and C/EBP [34,35]. CCAAT enhancer binding protein α (C/EBPα) is a member of the basic leucine zipper protein family to enhance transcriptional activity in the promoter region and plays an important role in petal development [35]. DNA methylation can regulate gene transcription and expression by inhibiting the ability of methylation-sensitive transcription factors to bind to DNA or binding repressor proteins to inhibit the binding of methylation non-sensitive transcription factors [36]. Therefore, we hypothesized that the methylation of mC-16 might cause the failure of transcription factor C/EBPα to bind to the target sequence, thereby inhibiting the expression of *CHI* gene and further affecting differential expression of the *CHI* gene in petal tissues during development. In the future, it is necessary to not only verify whether methylation modification of C/EBPα will affect its binding to the promoter DNA of *CHI* gene using electrophoretic mobility shift assays (EMSA), but also analyze the effect of *CHI* gene expression on the petal color of *P. lactiflora* using RNAi and overexpression approaches. In the present study, we revealed preliminarily the regulatory mechanism of *CHI* gene expression from the perspective of epigenetics, providing the theoretical references for further study on *CHI* gene function of *P. lactiflora* in the future.

## Supporting information

**Figure S1.** HPLC chromatograms of *Paeonia lactiflora* in petals. a. Anthocyanins, detected at 525 nm; b. Anthoxanthins, detected at 350 nm; a1–a2 indicate identifed anthocyanins; f1–f6 indicate identifed anthoxanthins.

**Table S1.** Retention time, ultraviolet-visible spectral properties, mass spectrometric data and tentative identification of the compounds detected in *Paeonia lactiflora* petals with control and Pro-Ca treatment.

**Table S2.** Basic BSP-Miseq sequencing data of all CpG sites in petals of *Paeonia lactiflora* at different developmental stages. S1, S2, S3, S4 represent the flower-bud, initiating bloom, bloom and withering stages, respectively. AV represents the average degree of methylation.

## Abbreviations

CHI: chalcone isomerase
BSP: bisulfite sequencing PCR
BST-DNA: bisulfite-treated DNA
EMSA: electrophoretic mobility shift assays
HPLC: high-performance liquid chromatograph
RACE: rapid-amplification of cDNA ends
