# Supplementary material for: Age-associated methylation change of CHI promoter in herbaceous peony (Paeonia lactiflora Pall)
Source: Biosci Rep. 2018 Sep 14;38(5):BSR20180482. doi: 10.1042/BSR20180482 (PMC6137250; doi:10.1042/BSR20180482)
Supplement: Supplementary file 1 [file bsr20180482_Supp1.pdf]

Supplementary Materials

Table S1. Retention time, ultraviolet-visible spectral properties, mass spectrometric data and tentative identification of the compounds detected in *Paeonia lactiflora* petals with control and Pro-Ca treatment.

Table S2. Basic BSP-Miseq sequencing data of all CpG sites in petals of *Paeonia lactiflora* at different developmental stages. S1, S2, S3, S4 represent the flower-bud, initiating bloom, bloom and withering stages, respectively. AV represents the average degree of methylation.

Figure S1. HPLC chromatograms of *Paeonia lactiflora* in petals. a. Anthocyanins, detected at 525 nm; b. Anthoxanthins, detected at 350 nm; a1–a2 indicate identifed anthocyanins; f1–f6 indicate identifed anthoxanthins.

Table S1: Retention time, ultraviolet-visible spectral properties, mass spectrometric data and tentative identification of the compounds detected in *P. lactiflora* petals with control and Pro-Ca treatment

Peak	Retention time (min)	Mass spectrometry information			Tentative identification	
		λ_{max} (nm)	$[M+H]^+$ (m/z)	MS^2 (m/z)		
a1	17.58	275; 515	611.15	449.04; 287.19	Cyanidin-3,5-di-*O*-glucoside	Cy3G5G
a2	25.27	275; 515	625.18	463.09; 301.11	Peonidin-3,5-di-*O*-glucoside	Pn3G5G
f1	19.09	260; 355	627.12	465.24; 303.13	Quercetin-3,7-di-*O*-glucoside	Qu3G7G
f2	25.04	265; 345	611.10	449.10; 287.28	Kaempferol-3,7-di-*O*-glucoside	Km3G7G
f3	37.45	265; 355	617.09	465.08; 303.09	Quercetin-3-*O*-galloylglucoside	Qu3GloG
f4	45.82	265; 355	464.99	303.01	Quercetin-3-*O*-glucoside	Qu3G
f5	53.44	270; 350	601.11	449.26; 287.21	Kaempferol-3-*O*-galloylglucoside	Km3GloG
f6	60.93	275; 345	449.02	287.09	Kaempferol-3-*O*-glucoside	Km3G

a1-a2 indicate identified anthocyanins; f1-f6 indicate identified anthoxanthins.

Sample	CpG1	CpG2	CpG3	CpG4	CpG5	CpG6	CpG7	CpG8
S1-1	64.1	49.97	61.68	63.26	69.84	64.17	62.14	51.05
S1-2	73.32	58.04	78.09	58.83	60.84	73.31	56.66	53.13
S1-3	69.04	62.94	89.71	69.14	71.62	85.78	29.14	36.51
S1-4	57.51	44.7	65.21	55.32	70.99	74.38	63.9	53.76
S1-5	68.56	57.55	69.91	74.87	51.45	24.52	95.45	52.96
S1-6	60.98	49.74	73.35	56.88	84.22	78.72	82.44	53.27
S1-7	81.3	70.11	57.2	60.81	86	61.81	78.64	30.99
S1-8	67.54	42.49	58.61	51.34	86.63	71.91	70.86	40.83
S2-1	63.75	49.01	77.6	55.26	71.57	66.74	70.3	48.37
S2-2	82.46	66.8	55.21	49.44	69.43	73.89	77.04	35.49
S2-3	65.41	49.09	68.32	59.26	75.58	74.8	64.51	47.18
S2-4	57.4	47.63	75.34	56.56	80.79	77.16	68.83	60.95
S2-5	64.54	58.58	65.77	45.35	70.61	51.39	50.57	53.32
S2-6	84.54	94.96	61.57	50.7	91.44	77.51	56.45	40.97
S2-7	73.76	54.17	62.51	74.71	70.25	64.72	72.03	37.2
S2-8	69.22	54.21	59.73	35.42	68.48	66.99	55.26	45.39
S3-1	64.7	50.82	74.35	62.91	69.19	65.21	61.18	64.43
S3-2	56.85	64	70.2	71.6	71.7	65.06	58.28	55.44
S3-3	78.89	57.13	74.01	68.47	59.38	53.25	45.4	69.98
S3-4	63.33	54.44	62.92	56.11	81.02	71.18	57.71	47.81
S3-5	77.3	59.88	67.77	70.66	62.94	49.45	50.95	54.69
S3-6	71.6	81.63	58.51	74.1	68.9	82.15	66.67	40.21
S3-7	69.56	57.56	68.34	63.24	63.88	78.74	62.67	49.66
S3-8	74.02	71.41	51.66	59.65	74.18	45.92	77.94	55.74
S4-1	77.93	64.52	67.32	80.25	54.46	45.37	62.46	51.44
S4-2	82.44	59.39	67.77	62.28	73.75	73.16	65.57	61.42
S4-3	62.78	72.11	64.97	65.22	70.1	69.04	66.94	59.31
S4-4	66.92	47.8	67.64	85.27	70.41	74.74	70.35	50.08
S4-5	72.45	62.58	71.28	75.63	67.36	72.7	55.09	71.51
S4-6	76.7	70.64	65.11	64.75	69.77	79.4	82.65	69.92
S4-7	86.71	62.99	70.71	73.18	69.86	76.16	72.16	61.4
S4-8	82.31	71.4	73.96	64.58	64.51	73.28	67.02	55.87

CpG9	CpG10	CpG11	CpG12	CpG13	CpG14	CpG15	CpG16	AV
57.83	64.25	34	47.1	75.33	73.88	56.28	60.67	60.10571
57.47	59.23	29.5	68.84	67.31	67.08	62.29	53.1	60.40571
68.36	55.9	30.82	58.17	51.72	58.37	48.93	56.18	57.88214
58.22	55.01	30.86	69.99	70.97	65.99	51.44	60.6	60.47429
47.89	39.02	11.19	48.25	68.21	56.89	64.18	66.53	55.09429
76.09	48.15	38.99	63.61	55.99	69.84	55.76	70.25	64.82571
56.02	65.45	30.32	40.85	76.79	57.75	67.51	75.69	60.41643
67.66	58.96	32.69	66.47	68.01	75.46	57.41	65.69	62.32357
62.96	61.11	37.25	76.28	73.44	63.6	59.06	45.43	62.06929
66.59	58.86	38.02	64.21	68.28	51.87	60.97	52.51	58.70071
56.13	56.01	34.11	69.24	59.66	71.43	55.73	58.79	60.76786
62.87	75.53	43.2	90.87	77.56	69.38	33.33	50.02	65.885
56.57	58.54	10.68	36.8	69.45	69.38	48.66	66.05	53.79571
59.09	45.26	37.44	74.15	67.67	64.75	47.36	48.1	58.74714
56.68	52.29	38.05	63.71	80.74	73.44	49.57	59.55	61.10357
52.4	59.78	43.78	55.48	74.86	70.21	64.21	53.66	57.54643
66.88	67.01	30.93	47.87	71.85	62.12	61.02	75.78	62.90929
62.65	66.54	22.87	50.75	70.7	66.65	56.09	50.93	59.96143
39.21	62.46	43.83	41.11	89.94	78.71	65.54	66.66	61.28214
56.98	57.46	31.58	70.53	73.47	72.18	52.8	80.43	62.29857
48.1	58.69	32.78	57.08	74.02	78.66	46.94	66.93	58.54714
62.43	59.95	35.8	67.11	75.71	69.21	54.31	67.08	63.01
61.43	59.16	33.91	58.98	80.52	80.05	58.7	69.41	63.47786
80.33	68.04	45.23	27.7	83.31	80.94	70.56	64.77	63.28357
63.59	69.81	34.05	55.78	81.36	65.1	55.93	78.32	61.80286
43.17	65.72	27.77	71.1	83.57	73.48	42.33	68.43	62.82286
50.23	62.81	30.5	61.4	85.05	65.82	51.66	75.2	62.73214
62.01	58.69	25.28	73.01	82.17	62.95	68.69	82.65	66.71
85.83	83.55	28.03	64.44	71.11	67.31	56.05	90.35	68.58857
78.14	79.66	47.36	72.63	83.36	51.1	47.84	85.29	69.78429
65.07	62.53	38.77	70.06	79.23	67.53	70.86	75.63	68.08214
52.52	58.96	21.7	59.85	79.56	82.73	77.23	86.76	65.60929

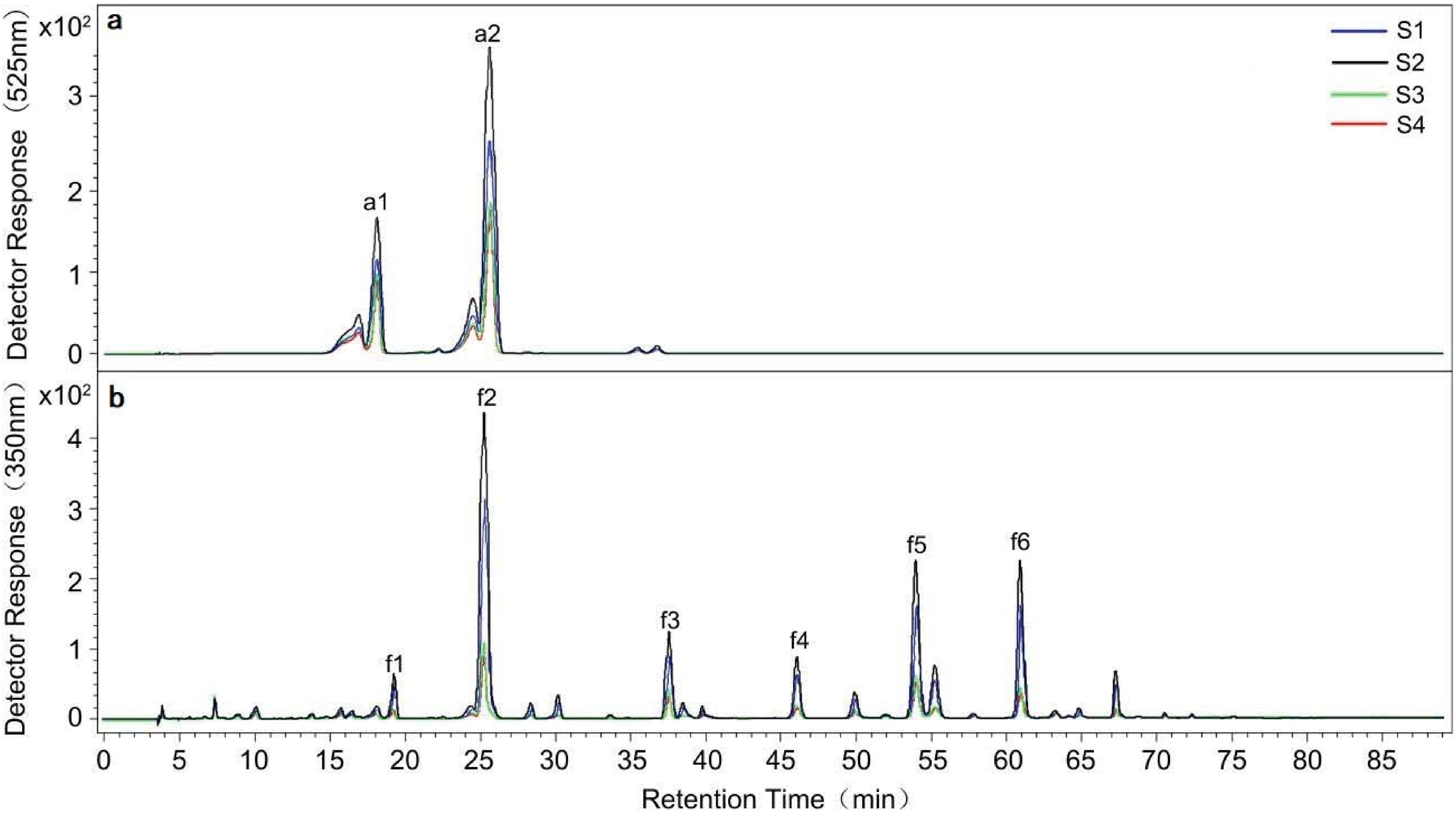

a
b
x10²
Detector Response（525nm）
Detector Response（350nm）
a1
a2
f1
f2
f3
f4
f5
f6
S1
S2
S3
S4
Retention Time（min）
